# Catechol-O-Methyltransferase (*COMT*) gene polymorphism and breast cancer risk in young women

**DOI:** 10.1054/bjoc.2001.2009

**Published:** 2001-09

**Authors:** M Bergman-Jungeström, S Wingren

**Affiliations:** Department of Biomedicine and Surgery, Division of Oncology, Faculty of Health Sciences, University Hospital, Linköping, S-581 85, Sweden

**Keywords:** Catechol-O-Methyltransferase, COMT, genetic polymorphism, breast cancer, early onset, catechol oestrogens

## Abstract

Oestrogen exposure has long been considered to be a main risk factor of breast cancer. More recently, interest has also focused on the possible carcinogenic influence from oestrogen metabolites, such as catechol oestrogens. O-methylation, catalysed by Catechol-O-Methyltransferase (COMT), is one pathway by which the potentially carcinogenic catechol oestrogens can be inactivated. The gene coding for COMT protein contains a single-nucleotide polymorphism (SNP), resulting in an amino acid shift Val→Met, which has been shown to determine high- and low-activity configuration of the enzyme. We hypothesized that the low-activity allele, COMT^Met^, may be implicated in early onset breast cancer. In the present case–control study, including 126 young breast cancer patients (≤ 36 years) and 117 healthy female blood donors, we analysed the association between COMT^Met^ genotype and risk of breast cancer. No significant difference in the frequency of low-/high-activity alleles was found between cases and controls, indicating that the polymorphism, as a single factor, may not contribute to breast carcinogenesis in young women. © 2001 Cancer Research Campaignhttp://www.bjcancer.com


*British Journal of Cancer* (2001) **85**(6), 859–862
© 2001 Cancer Research Campaign
doi: 10.1054/ bjoc.2001.2009, available online at http://www.idealibrary.com on IDEAL
http://www.bjcancer.com

### Catechol-O-Methyltransferase (*COMT*) gene polymorphism and breast cancer risk in young women

**M Bergman-Jungeström and S Wingren**

Department of Biomedicine and Surgery, Division of Oncology, Faculty of Health Sciences, University Hospital, S-581 85 Linköping, Sweden

**Summary** Oestrogen exposure has long been considered to be a main risk factor of breast cancer. More recently, interest has also focused on the possible carcinogenic influence from oestrogen metabolites, such as catechol oestrogens. O-methylation, catalysed by Catechol-O-Methyltransferase (COMT), is one pathway by which the potentially carcinogenic catechol oestrogens can be inactivated. The gene coding for COMT protein contains a single-nucleotide polymorphism (SNP), resulting in an amino acid shift Val→Met, which has been shown to determine high- and low-activity configuration of the enzyme. We hypothesized that the low-activity allele, $COMT^{Met}$, may be implicated in early onset breast cancer. In the present case–control study, including 126 young breast cancer patients (≤ 36 years) and 117 healthy female blood donors, we analysed the association between $COMT^{Met}$ genotype and risk of breast cancer. No significant difference in the frequency of low-/high-activity alleles was found between cases and controls, indicating that the polymorphism, as a single factor, may not contribute to breast carcinogenesis in young women. © 2001 Cancer Research Campaign http://www.bjcancer.com

**Keywords**: Catechol-O-Methyltransferase; COMT; genetic polymorphism; breast cancer; early onset; catechol oestrogens

Oestrogen exposure has been considered to be one of the main risk factors for breast cancer. One characteristic of oestrogen is its mitogenic action in hormone-sensitive tissues such as uterus and breast. More recently, interest has also focused on the possible carcinogenic influence from oestrogen metabolites. These metabolites may mediate their effect by; activation of the classical oestrogen receptor, interaction with other specific receptors or effectors, direct binding to DNA, or by other mechanisms (reviewed by Zhu and Conney, 1998). Among the oestrogen metabolites, the two catechol oestrogens, 2-hydroxyoestradiol (2-$OHE_2$) and 4-hydroxyoestradiol (4-$OHE_2$), have shown several biological effects (Weisz, 1994; Liehr, 1997; Zhu and Conney, 1998). The 4-hydroxyolated metabolite binds to and activates the oestrogen receptor with approximately the same affinity as oestradiol. However, the interaction with the hormone receptor is markedly reduced for the 2-$OHE_2$, which therefore may possess a weaker hormonal potency as compared with the parent hormone; oestradiol (Liehr, 1997; Zhu and Conney, 1998). Both animal models and in vitro studies (reviewed by Liehr, 1997) have shown that 4-$OHE_2$ promotes cell proliferation and carcinogenesis. Furthermore, Liehr and Ricci (1996) observed elevated 4-$OHE_2$ levels in human breast cancer tissue as compared to normal breast tissue and in addition, both 2- and 4-$OHE_2$ have been reported to undergo metabolic oxidation to the chemically highly reactive oestrogen-derived semiquinones and quinones (Liehr and Yager, 1996; Cavalieri et al, 1997). These metabolites are possible candidates as agents in the carcinogenic process. Their interaction with DNA may cause formation of adducts and furthermore in the semiquinones/quinones redox cycling processes, reactive oxygen species such as superoxide are formed (Cavalieri et al, 1997; Zhu and Conney, 1998). Free radicals have been demonstrated to damage both DNA and other cellular constituents and such damage seems likely to be an important event in the aetiology of human cancers (Dreher and Junod, 1996; Marnett, 2000). One of the major inactivation pathways of 2- and 4-$OHE_2$ is through O-methylation to 2- and 4-methoxyoestradiol (Yager and Liehr, 1996; Zhu and Conney, 1998). These metabolites are more lipophilic, have longer half-lives than their corresponding $OHE_2$, and they have a weak or no binding affinities to the classical oestrogen receptor (Zhu and Conney, 1998). Interestingly, 2-methoxyestradiol (2-$MeE_2$) has been reported to influence carcinogenesis by inhibition of endothelial cell proliferation, and migration and angiogenesis (Fotsis et al, 1994).

The O-methylation of 2- and 4-$OHE_2$ is catalysed by the enzyme Catechol-O-Methyltransferase (COMT) (Yager and Liehr, 1996). COMT activity is highly present in liver and kidney, and it is also found at significant levels in brain, red blood cells, uterine endometrium and the mammary gland. The *COMT* gene, located on chromosome 22q11.1–q11.2, contains a single-nucleotide polymorphism (G→A) in codon 158/108 of the membrane bound/cytosolic form (Butterworth et al, 1996). The single-nucleotide transition results in an amino acid change, Val→Met, which has been shown to determine high- and low-activity alleles of the enzyme (Lachman et al, 1996). The $COMT^{Met}$ allele, coding for the low-activity and heat-labile enzyme, has proved to be 4- to 5-fold less effective in methylating catechol substrates in vitro (Scanlon et al, 1979). A possible accumulation of 4-$OHE_2$, caused by decreased COMT activity is hypothesized to confer increased risk of breast cancer.

The aetiology of breast cancer in young women has shown differences in terms of inheritance, carcinogenesis and prognosis compared to their older counterparts (Nixon et al, 1994; Bonnier et al, 1995), possibly indicating separate biological origin of the disease. Besides germ-line mutations in the breast cancer susceptibility genes (i.e., *BRCA1* and *BRCA2*), little is known about other factors implicated in breast carcinogenesis among young women.

*Received 16 February 2001*
*Revised 20 June 2001*
*Accepted 2 July 2001*

*Correspondence to:* M Bergman-Jungeström

Most of the current risk factors, including age at menarche and menopause, age at first full-time pregnancy as well as the number of parturitions, are indicators of cumulative oestrogen exposure. The cellular levels of oestrogen and its metabolites may be controlled through both genetic and environmental factors. We and others have hypothesized that polymorphisms in crucial genes, e.g. genes coding for enzymes involved in oestrogen metabolism, may be predisposing for breast cancer. In a previous investigation, of one SNP in the *CYP17* gene, done on the same populations as the present study, a significant association between genetic variants and breast cancer risk was found (Bergman-Jungeström et al, 1999). Also, other studies of genetic polymorphism in genes involved in the biosynthesis or metabolism of endogenous and exogenous carcinogens have shown association to altered breast cancer risk and tumour progression (Zhong et al, 1993; Taioli et al, 1995; Lundin et al, 1999; Sprudle et al, 2000).

The intention with the present study was to investigate the conceivable association between the low-activity *COMT* gene and risk of early onset breast cancer. We analysed frequencies of the Val/Met alleles in a group of young breast cancer patients and compared with a control population consisting of young healthy, female blood donors.

### MATERIALS AND METHODS

### Study population

The study included 126 young breast cancer patients diagnosed in the South-East Sweden Health Care Region between 1980 and 1994. At the time of diagnosis, the patients were between 22 and 36 years of age, with a median age of 34 years. Tissue samples from archival material were obtained from the pathology departments of hospitals in Linköping, Norrköping, Jönköping and Kalmar. The control group included 117 healthy female blood donors who were between 18 and 39 years of age (median age 30 years). Blood from the control population was collected in EDTA tubes.

### Methods

A microdissection technique was used to prevent contamination with malignant cells. A 5-μm section from each paraffin block of axillary lymph nodes was stained with haematoxylin-eosin, and areas comprises normal cells were selected by a pathologist. The selected areas were then identified in the 30-μm sections and collected, before preparation of DNA. The paraffin was removed by repeated extractions with xylene, followed by hydration in decreasing concentrations of ethanol. The tissue was then digested in a proteinase K solution at 55˚C for 36 hours, and by-products were subsequently extracted with phenol, phenol-chloroform (1:1), and chloroform. The nucleic acids were precipitated in 95% ethanol containing sodium acetate (0.1 M) for approximately 1 hour at –20˚C and thereafter pelleted by centrifugation at 12 000 ***g***. The DNA pellet was washed in 70% ethanol, vacuum dried and then resuspended in water.

Samples from the control population were purified using a rapid DNA preparation method (Wizard Genomic DNA Purification System, Promega). Erythrocytes were lysed before the leucocytes were isolated by centrifugation. The leucocytes were then suspended in a nuclei lysis solution, and proteins were precipitated and removed by centrifugation. Thereafter, the DNA was precipitated in isopropanol. The pelleted DNA was washed with 70% ethanol, dried and finally resuspended in 10 mM Tris-HCl/1 mM EDTA (pH 4.0). The concentration of DNA, from both cases and controls, was determined by spectrophotometry.

### Identification of and screening for A1/A2 alleles

Polymerase chain reaction (PCR) amplification of a 237 base-pair DNA fragment of the *COMT* gene, including the part of exon 4 containing the polymorphism, was performed using the forward primer COMT E4U-2 (5´-TACTGTGGCTACTCAGCTGTGC-3´) and the reverse primer COMT E4L-2 (5´-GTGAACGTGGTGTGAACACC-3´) (Lavigne et al, 1997). PCR reactions were carried out in 21 μl aliquots containing 25–50 ng of genomic DNA, primers (each 1.0 μM), 1 × reaction buffer, dNTPs (each 200 μM) and 1 unit of *Taq* polymerase (Promega). We performed 30–40 cycles of PCR amplification, with denaturation at 93˚C for 45 s, annealing at 55˚C for 1 min, and extension at 72˚C for 1 min. An initial 3 min denaturation step at 94˚C and a final 4 min extension at 72˚C were used. The PCR products were incubated with the restriction enzyme Nla III (New England Biolabs) for 3 h at 37˚C. The enzyme was subsequently denatured at 60˚C for 20 min and the amplicons were separated by agarose (3%) gel electrophoresis. The gel was stained with ethidium bromide and the DNA was visualized in UV light. Restriction products of 27, 42 and 54 base pairs were present in every sample. The presence of a G at position 1947 ($COMT^{Val}$ allele) generates a unique 114 base-pair fragment, while the occurrence of an A ($COMT^{Met}$ allele) resulted in digestion of the 114 base-pair fragment into products of 96- and 18-base pairs respectively (Figure 1).

### Statistical methods

Odds ratios (OR) were calculated to estimate the risk of breast cancer and *P* values were calculated by $\chi^2$-analysis. Furthermore, $\chi^2$ test was also used to investigate whether the control population was consistent with the Hardy–Weinberg equilibrium.

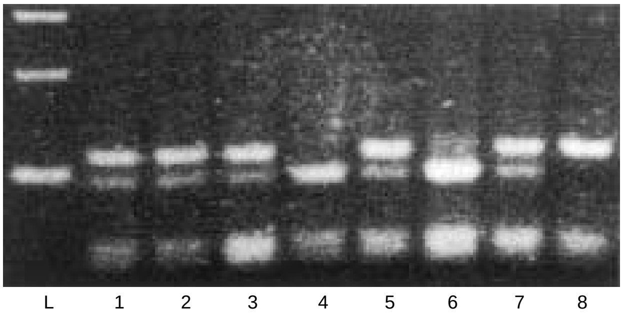

**Figure 1** Nla III restriction fragments of COMT alleles separated by agarose gel electrophoresis. The presence of a G at position 1947 ($COMT^{Val}$ allele) generates a unique 114 base pair fragment, while the occurrence of an A ($COMT^{Met}$ allele) results in digestion of the 114 base pair fragment into products of 96- and 18-base pairs respectively. Line L contains a 100-base pair ladder. Lane 4 and 6 show a homozygous low activity allele ($COMT^{Met}$/$COMT^{Met}$, lane 8 the homozygous high-activity allele ($COMT^{Val}$/$COMT^{Val}$) and lane 1, 2, 3, 5 and 7 the heterozygous pattern

 *© 2001 Cancer Research Campaign*

### RESULTS

Odds ratios were calculated to evaluate the risk of breast cancer for individuals homozygous or heterozygous for the low-activity allele, $COMT^{Met}/COMT^{Met}$, $COMT^{Val}/COMT^{Met}$ and $COMT^{Met}/COMT^{Met}$ + $COMT^{Val}/COMT^{Met}$, compared to subjects who were homozygous for the high-activity allele; $COMT^{Val}/COMT^{Val}$. The frequencies of the COMT genotype among the breast cancer patients and the control population are shown in Table 1 in addition to the calculated odds ratios and *P* values. We found no increased breast cancer risk among low-activity allele carriers compared to individuals who were homozygous for the $COMT^{Val}$ allele. The $COMT^{Met}/COMT^{Met}$ carriers had an OR of 0.87, 95% confidence interval (CI), 0.34–2.2, *P* = 0.74. OR among the heterozygous women was 0.85; CI: 0.35–2.1, *P* = 0.70, and when $COMT^{Met}/COMT^{Met}$ + $COMT^{Val}/COMT^{Met}$ alleles were considered together the OR was estimated to 0.86; (CI), 0.37–2.00, *P* = 0.70. Moreover, the genotype dispersal in the control population was within that expected according to the Hardy–Weinberg equilibrium.

### DISCUSSION

It has been hypothesized that women, homo-or heterozygous for the $COMT^{Met}$ allele, may exhibit lower COMT activity in the breast. Decreased COMT activity may result in an accumulation of potentially carcinogenic oestrogen metabolites, and therefore cause a higher risk of breast cancer. This has been tested in both post-and premenopausal women by several investigators (Lavigne et al, 1997; Thompson et al, 1998; Millikan et al, 1998; Huang et al, 1999) but the results are discordant. In the present study, comprising early-onset breast cancer patients and a control population of young blood donors, Val/Met polymorphism revealed no association with altered breast cancer risk. This finding is consistent with that reported by Millikan et al (1998), who performed a population-based case–control study including 654 invasive breast cancer cases and 642 controls of both pre- and postmenopausal women. No association between one or more copies of the low-activity COMT allele and breast cancer risk was found. A similar result was obtained by Thompson et al (1998), when risk was estimated in a case–control study consisting of both pre- and postmenopausal subjects in a study based on 281 breast cancer patients and 289 controls. Nevertheless, when the study population was stratified according to menopausal status, the $COMT^{Met}$ allele was associated with breast cancer risk among premenopausal individuals but, interestingly they found an inverse association in postmenopausal women. In contrast to the results obtained by Thompson et al, Lavigne and co-workers (1997) reported a trend towards increased breast cancer risk in postmenopausal women homozygous for the $COMT^{Met}$ allele and further, their results slightly indicate a decreased risk for the premenopausal subjects. A recent Taiwanese population case–control study, in which 150 patients and 150 controls were examined, revealed a significant increase in breast cancer risk associated to the $COMT^{Met/Met}$ genotype (Huang et al, 1999). The homozygous pattern of the $COMT^{Met}$ allele was associated with higher risk in the postmenopausal women compared to their premenopausal counterparts. However, it should be noted that the number of participants with $COMT^{Met/Met}$ genotype were few.

Earlier studies of the *COMT* gene polymorphism, investigating its possible role as a modifier of breast carcinogenesis, indicate the importance of considering other parameters closely related to oestrogen exposure (Lavigne et al, 1997; Millikan et al, 1998; Thompson et al, 1998; Huang et al, 1999). One such parameter is body mass index (BMI), which has been shown to affect oestrogen exposure, possibly as a result of aromatization of androgens to oestrogens in fat tissue (Ackerman et al, 1981). Body mass index has been noted to influence breast cancer risk in several studies, and this effect may differ depending on menopausal status. Postmenopausal adipose women have been reported to be at higher risk of breast cancer compared to their leaner counterparts, while a decreased risk has been observed in premenopausal women with high BMI (Harris et al, 1992; Magnusson et al, 1998; van den Brandt, 2000). Most of the cases in the present study were diagnosed in the 1980s and data about BMI were not collected; consequently information about BMI is not available. Considering the combination of BMI and COMT genotype, the results are mainly compatible. Lavigne et al (1997) found that high BMI, in combination with the homozygous low-activity COMT genotype, significantly increased breast cancer risk (OR, 3.6) in postmenopausal women. Thompson and co-workers (1998) demonstrated that high BMI and presence of at least one $COMT^{Met}$ allele was associated with a significant breast cancer risk in premenopausal women, but no such correlation among the postmenopausal women was shown.

In summary, there are several reports indicating an influence from oestrogen metabolites on cellular properties closely related to carcinogenesis. A reduced COMT activity may result in enhanced cell proliferation and increased formation of free radicals as a consequence of a possible accumulation of 4-$OHE_2$ and furthermore a decreased inhibition of angiogenesis due to less synthesis of 2-$MeE_2$. Yet, the present case–control study examining young breast cancer patients and age-matched controls, did not show any increased breast cancer risk associated with the low-activity COMT genotype. Our result indicates that the investigated genetic

**Table 1** Calculated odds ratios (ORs), COMT genotype- and allele frequencies for young breast cancer patients and a control population

| Genotype | Control (%) (*n* = 117) | Cases (%) (*n* = 126) | OR | 95% CI | *P* |
|---|---|---|---|---|---|
| $COMT^{Val/Val}$ | 13 (11) | 16 (13) | 1.0 | | |
| $COMT^{Val/Met}$ | 61 (52) | 64 (51) | 0.85 | 0.35–2.1 | 0.70 |
| $COMT^{Met/Met}$ | 43 (37) | 46 (36) | 0.87 | 0.34–2.2 | 0.74 |
| $COMT^{Val/Met}$ + $COMT^{Met/Met}$ | 104 (89) | 110 (87) | 0.86 | 0.37–2.0 | 0.70 |
| Allele frequencies | | | | | |
| $COMT^{Val}$ | 87 (37) | 96 (41) | | | |
| $COMT^{Met}$ | 147 (58) | 156 (62) | | | |

$COMT^{Val}$ = high activity allele; $COMT^{Met}$ = low activity allele.

© 2001 Cancer Research Campaign variant of COMT may not alone be an important risk factor in young women. Polymorphisms in crucial genes are probably associated with a more modest breast cancer risk as compared to the highly penetrant germ-line mutations in breast cancer susceptibility genes (e.g., *BRCA1* and *BRCA2*). Nevertheless, genetic polymorphisms are prevalent in the general population and may therefore explain a greater proportion of breast cancer than mutations in the highly penetrant genes (Weber and Nathanson, 2000). Genetic variants in combination with multiple endogenous and exogenous factors may significantly increase breast cancer risk.

### ACKNOWLEDGEMENTS

We thank the Departments of Pathology at the Hospitals in Linköping, Norrköping, Jönköping and Kalmar for kindly providing archival material. This work was supported by The Research Council in South East Sweden and The Swedish Cancer and Allergy Society.

### REFERENCES

Ackerman GE, Smith ME, Mendelson CR, MacDonald PC and Simpson ER (1981) Aromatization of androstenedione by human adipose tissue stromal cells in monolayer culture. *J Clin & Metab* **53**: 412–417

Bergman-Jungeström M, Gentile M, Lundin AC, the South-east breast cancer group and Wingren S (1999) Association between CYP17 gene polymorphism and risk of breast cancer in young women. *Int J Cancer (Pred Oncol)* **84**: 350–353

Bonnier P, Romain S, Charpin C, Lejeune C, Tubiana N, Martin PM and Piana L (1995) Age as a prognostic factor in breast cancer: relationship to pathology and biological features. *Int J Cancer* **62**: 138–144

Cavalieri EL, Stack DE, Devanesan PD, Todorovic R, Dwivedy I, Higginbotham S, Johansson SL, Patil KD, Gross ML, Gooden JK, Ramanathan RL and Rogan EG (1997) Molecular origin of cancer: Catechol estrogen-3,4-quinones as endogenous tumor initiators. *Proc Natl Acad Sci* **94**: 10937–10942

Dreher D and Junod AF (1996) Role of oxygen free radicals in cancer development. *Eur J Cancer* **32A**: 30–38

Fotsis T, Zhang Y, Pepper MS, Adlercreutz H, Montesano R, Nawroth PP and Schweigerer L, (1994) The endogenous oestrogen metabolite 2-methoxyoetradiol inhibits angiogenesis and suppresses tumour growth. *Nature* **368**: 237–239

Harris JR, Lippman ME, Veronesi U and Willett W (1992) Breast cancer. *N Engl J Med* **327**: 319–328

Huang CS, Chern HD, Chang KJ, Cheng CW, Hsu SM and Shen CY (1999) Breast cancer risk associated with genotype polymorphism of the estrogen-metabolizing genes CYP17, CYP1A1, and COMT: a multigenic study on cancer susceptibility. *Cancer Res* **59**: 4870–4875

Lachman HM, Papolos DF, Saito T, Yu YM, Szumlanski CL and Weinshilboum RM (1996) Human catechol-o-methyltransferase pharmacogenetics: description of a functional polymorphism and its potential application to neuropsychiatric disorders. *Pharmacogenetics* **6**: 243–250

Lavigne JA, Helzlsouer KJ, Huang HY, Strickland PT, Bell DA, Selmin O, Watson MA, Hoffman S, Comstock GW and Yager JD (1997) An Association between the allele coding for a low activity variant of catechol-o-methyltransferase and the risk for breast cancer. *Cancer Res* **57**: 5493–5497

Li SA, Purdy RH and Li JJ (1989) Variations in catechol O-methyltransferase activity in rodent tissues: possible role in estrogen carcinogenicity. *Carcinogenesis* **10**: 63–67

Liehr JG (1997) Dual role of oestrogens as hormones and pro-carcinogens: tumour initiation by metabolic activation of oestrogens. *Eur J Cancer Prev* **6**: 3–10

Liehr JG and Ricci MJ (1996) 4-Hydroxylation of estrogens as marker of human mammary tumors. *Proc Natl Acad Sci* **93**: 3294–3296

Lundin AC, Söderkvist P, Eriksson B, Bergman-Jungeström M, Wingren S and the south-east Sweden Breast Cancer Group (1999) Association of breast cancer progression with a vitamin D receptor gene polymorphism. *Cancer Res* **59**: 2332–2334

Magnusson C, Baron J, Persson I, Wolk A, Bergström R, Trichopoulos and Adami H-O (1998) Body size in different periods of life and breast cancer risk in post-menopausal women. *Int J Cancer* **76**: 29–34

Marnett LJ (2000) Oxyradicals and DNA damage. *Carcinogenesis* **21**: 361–370

Millikan RC, Pittman GS, Tse CKJ, Duell E, Newman B, Savitz D, Moorman PG, Boissy RJ and Bell DA (1998) Catechol-o-methyltransferase and breast cancer risk. *Carcinogenesis* **19**: 1943–1947

Nixon AJ, Neuberg D, Hayes DF, Gelman R, Connolly JL, Schnitt S, Abner A, Recht A, Vicini F and Harris JR (1994) Relationship of patient age to pathologic feature of the tumor and prognosis for patients with stage I or II breast cancer. *J Clin Oncol* **12**: 888–894

Scanlon PD, Raymond FA and Weinshilboum RM (1979) Catechol-O-methyltransferase: thermolabile enzyme in erythrocytes of subjects homozygous for allele for low activity. *Science* **5**: 63–65

Sprudle AB, Hopper JL, Dite GS, Chen X, Cui J, McCredie MRE, Giles GG, Southey MC, Venter DJ, Easton DF and Chenevix-Trench G (2000) CYP17 promoter polymorphism and breast cancer in Australian women under age forty years. *J Nat Cancer Inst* **92**: 1674–1681

Taioli E, Trachman J, Chen X, Toniolo P and Garte SJ (1995) A CYP1A1 Restriction fragment length polymorphism is associated with breast cancer in African-American women. *Cancer Res* **55**: 3757–3758

Thompson PA, Shields PG, Freudenheim JL, Stone A, Vena JE, Marshall JR, Graham S, Laughlin R, Nemoto T, Kadlubar FF and Ambrosone CB (1998) Genetic polymorphism in catechol-o-methyltransferase, menopausal status and breast cancer risk. *Cancer Res* **58**: 2107–2110

van den Brandt PA, Spiegelman D, Yaun SS, Adami HO, Beeson L, Folsom AR, Fraser G, Goldbohm RA, Graham S, Kushi L, Marshall JR, Miller AB, Rohan T, Smith-Warner SA, Speizer FE, Willett WC, Wolk A and Hunter DJ (2000) Pooled analysis of prospective cohort studies on height, weight, and breast cancer risk. *Am J Epidemiol* **15**: 514–527

Weber BL and Nathanson KL (2000) Low penetrance genes associated with increased risk for breast cancer. *Eur J Cancer* **36**: 1193–1199

Weisz J (1994) Biogenesis of catecholestrogens: A mechanism for metabolic activation of estrogens. *Polycyclic Aromatic Compounds* **6**: 241–251

Yager JD and Liehr JG (1996) Molecular mechanisms of estrogen carcinogenesis. *Annu Rev Pharmacol Toxicol* **36**: 203–232

Zhong S, Wyllie AH, Barnes D, Wolf CR and Spurr NK (1993) Relationship between the GSTM1 genetic polymorphism and susceptibility to bladder, breast and colon cancer. *Carcinogenesis* **14**: 1821–1824

Zhu BT and Conney AH (1998) Functional role of estrogen metabolism in target cells: review and perspectives. *Carcinogenesis* **19**: 1–27

 *© 2001 Cancer Research Campaign*
